# Emergence of Intronless Evolutionary Forms of Stress Response Genes: Possible Relation to Terrestrial Adaptation of Green Plants

**DOI:** 10.3389/fpls.2019.00083

**Published:** 2019-02-07

**Authors:** Sergey Y. Morozov, Andrey G. Solovyev

**Affiliations:** ^1^A. N. Belozersky Institute of Physico-Chemical Biology, Moscow State University, Moscow, Russia; ^2^Department of Virology, Biological Faculty, Moscow State University, Moscow, Russia; ^3^Institute of Molecular Medicine, Sechenov First Moscow State Medical University, Moscow, Russia

**Keywords:** stress-related genes, plant-specific 4/1 gene, intronless gene, evolution, charophytes, Klebsormidium, chara

The currently known gene repertoire related to terrestrialization of plants includes genes of responses to different stresses, such as UV radiation, microbial pathogen attacks, desiccation, salinity, low, and high temperature. Evolutionary details of exon-intron organization in these genes are not analyzed comprehensively, although such an analysis would be rather informative and important for understanding their evolutionary history. Particularly, a series of papers on Phragmoplastophyta stress-related intron-containing genes show their origin by horizontal gene transfer of intronless prokaryotic genes (Emiliani et al., 2009; Fang et al., 2017).

The 4/1 protein is specific to plants; biochemical and cell biology data were obtained mostly for the *Nicotiana tabacum* 4/1 protein (Nt-4/1) (Makarova et al., 2011; Morozov et al., 2014; Atabekova et al., 2018). Recently, we reported that Nt-4/1 could respond to mechanical and temperature stresses by re-localization into numerous small spherical bodies likely associated with the cortical ER-plasma membrane contact sites (Atabekova et al., 2018). Previous studies have shown that there are some potential stress-responsive proteins among two dozen Arabidopsis and tobacco proteins interacting with 4/1 in the yeast two-hybrid system. These proteins include PBL, the ortholog of the well-characterized human ER-localized BAP31 protein; CPL, the phosphatase known to dephosphorylate the C-terminal domain of the largest subunit of DNA-dependent RNA polymerase II and involved in responses to different abiotic stress; and several stress-related transcription factors (Minina et al., 2009; Atabekova et al., 2017, 2018; Pankratenko et al., 2017). Importantly, the data available in public domain microarray databases showed that expression levels of Arabidopsis and rice 4/1 genes could significantly change in response to several stress factors, particularly, anoxia, and fungal infection (Solovyev et al., 2013a; Morozov et al., 2014).

According to computer predictions, the secondary structure of myosin-like Nt-4/1 protein is mainly α-helical; Nt-4/1 was predicted to have six extended α-helices, and five of them represented coiled-coil structural elements (von Bargen et al., 2001; Makarova et al., 2011, 2014; Solovyev et al., 2013b) (Supplementary Figure 1). While silencing of 4/1 gene in transgenic *N. benthamiana* caused no major morphological alterations in plants (Makarova et al., 2011; Morozov et al., 2014), transient virus-induced silencing of 4/1 gene in *N*. *benthamiana* resulted in faster phloem transport of *Potato spindle tuber viroid* (Solovyev et al., 2013a). These data were in line with our findings showing that at the 4/1 promoter is active in veins, with its activity being detected mostly in xylem parenchyma, phloem parenchyma, and primary phloem cells (Solovyev et al., 2013a). Organ- and tissue-specific expression patterns of some other plant 4/1 genes in public microarray databases were found to be consistent with 4/1 expression in conductive tissues (Solovyev et al., 2013a; Morozov et al., 2014). Thus, we proposed that the 4/1 protein could influence stress signaling in the vasculature (Atabekova et al., 2018).

The 4/1 genes encoded by the genomes of genera *Arabidopsis* and *Nicotiana* were shown to contain eight exons and seven introns (Paape et al., 2006; Makarova et al., 2011). A similar exon-intron structure was found for most 4/1 genes encoded by other dicotyledonous and monocotyledonous plants with some notable exceptions (Morozov et al., 2015) (Figure 1). To better understand the evolution of 4/1 genes, a comparative analysis of these genes in lower land plants in combination with structural analysis of encoded proteins was carried out. We have partially or completely sequenced 4/1 genes of some representatives of liverworts, Lycopodiopsida, ferns, and gymnosperms (Morozov et al., 2015). Interestingly, using publicly available databases containing next generation sequencing data, we were unable to identify 4/1-specific signatures in genomes of mosses *Physcomitrella patens* (class Bryopsida) and *Sphagnum fallax* (class Sphagnopsida). However, we reported that the genomes of basal land plants *Marchantia polymorpha* (class Marchantiopsida) and *Anthoceros agrestis* (class Anthocerotopsida) as well as partial assembled charophyte cDNA sequences from *Chaetosphaeridium globosum* (class Coleochaetophyceae) and several species of class Zygnematophyceae (including *Spirogyra pratensis*), could encode proteins similar to 4/1 proteins of Magnoliophyta (Morozov et al., 2014, 2015). Importantly, our efforts to reveal 4/1-like genes in basal charophyte classes Klebsormidiophyceae (Hori et al., 2014) and Chlorokybophyceae as well as in all moss classes, failed (Morozov et al., 2015).

**Figure 1:**
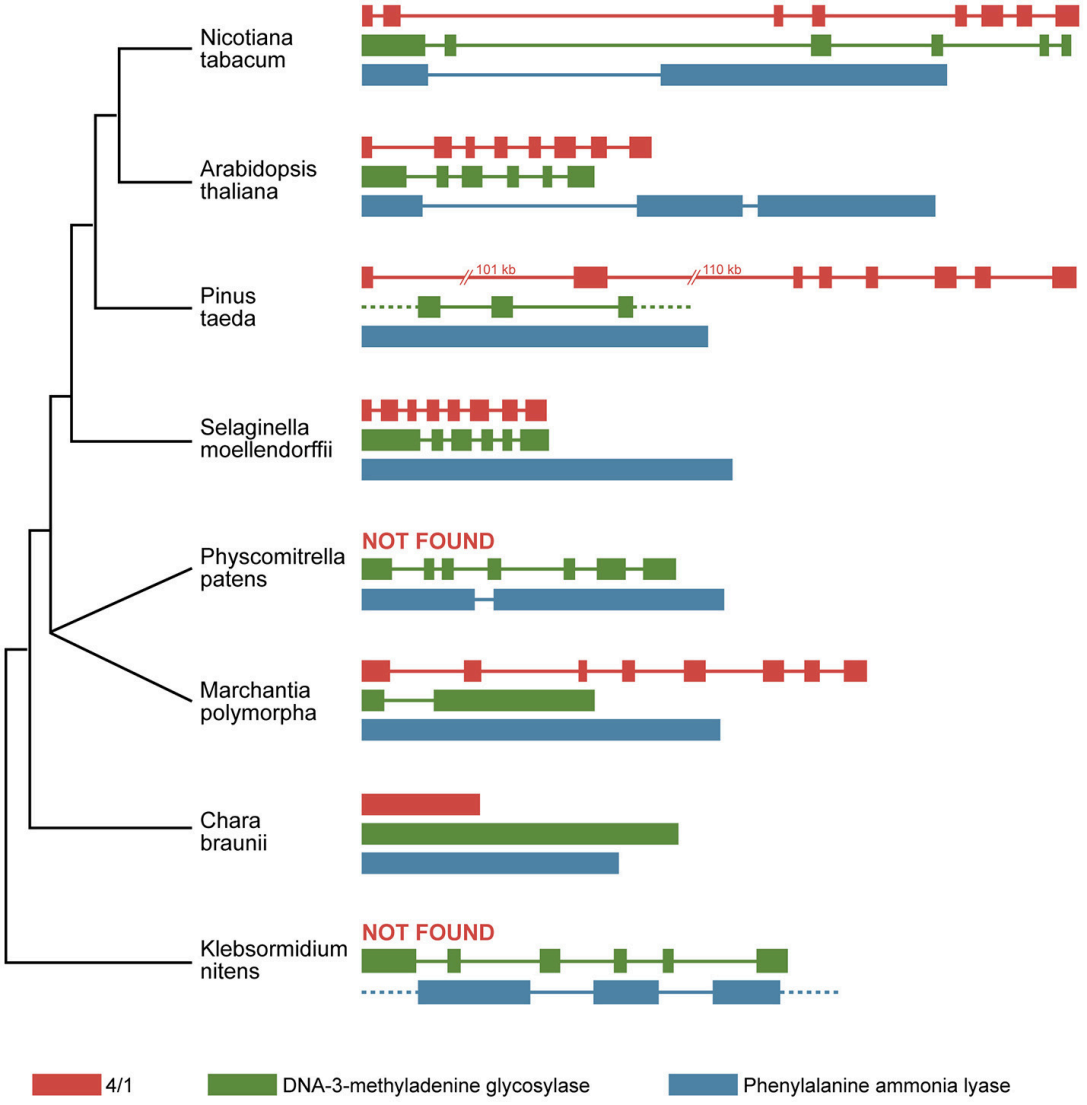
The exon/intron structure of selected plant stress-related genes in representatives of different taxa. Exons are indicated by boxes, and introns are indicated by lines. A phylogenetic synopsis for presented plant species is shown on the left. Only two classes of charophytes are shown (Klebsormidium nitens–class Klebsormidiophyceae; Chara braunii–class Charophyceae). The representatives of two basal classes Mesostigmatophyceae and Chlorokybophyceae as well as two classes closest to land plants (Coleochaetophyceae and Zygnematophyceae), are not shown because of the lack of genomic sequence data.

Recently, a nearly complete genomic sequence has been reported for *Chara braunii* (class Charophyceae) (Nishiyama et al., 2018). This genome encodes the 4/1-like protein protein CBR g25877 (Supplementary Figure 1) and, most interestingly, the revealed genomic 4/1-like locus of *C. braunii* contains no introns. Why the *Chara* 4/1-like gene is intronless, whereas almost all known 4/1 genes in Magnoliophyta and lower land plants contain introns (Figure 1) (Morozov et al., 2015)? We propose that a presumed precursor 4/1-like gene in basal charophytes initially originated by capturing an alpha-helical myosin-like cistron by a retrotransposon and further retrotransposon-dependent transfer to the algal genome. The intronless 4/1-like gene could appear directly in class Charophyceae. Indeed, *C. braunii* genome contains up to 75% repetitive elements, some of which represent retrotransposons (Nishiyama et al., 2018). Potentially, retrotransposition appears to enrich the genome with large numbers of duplicate retrocopies that can act as a source for both new composite genes in the same species and new genomic sequences in foreign species. It should be noted that acquisition of intronless genes could occur without a reverse transcription event, possibly by introgression of bacterially encoded sequences in plant genomes, as it has been suggested in several studies (Emiliani et al., 2009; Yue et al., 2012; Yang et al., 2014; Hu et al., 2016; Fang et al., 2017).

What could be the primary fuction(s) and the origin of intronless genes in the genome of *C. braunii*? Assuming our recent data on the involvement of Nt-4/1 protein into stress responses (Atabekova et al., 2018), we hypothesize that acquisition and fixation of 4/1-like genes, as well as other stress-related genes, in charophyte genomes might contribute to terrestrial adaptation of green plants subjected to many new stress factors such as brighter sunlight (UV radiation), lack of efficient support against gravity, dehydration, and high carbon dioxide levels in the atmosphere (Fang et al., 2017; Pierangelini et al., 2017, 2018). In fact, *C. braunii* genome is found to encode a number of evolutionary novel, compared to aquatic green algae, genes related to plant terrestrialization, particularly, genes responsible for reactive oxygen species scavenging (Nishiyama et al., 2018).

Another possibility is that the acquisition of intron-containing stress-related genes upon terrestrial adaptation could involve reverse transcription that likely occurred during a stress-dependent retrotransposition burst when transcription of genes involved in stress response is up-regulated and the levels of reverse transcriptase encoded by LTR retrotransposons are high (Cavrak et al., 2014; Ito et al., 2016; Gaubert et al., 2017; Masuta et al., 2018). In favor of the latter hypothesis, some plant stress-related genes are presented by intronless copies in the *C. braunii* genome and intron-containing copies in the genomes of *Klebsormidium* genus, a more basal species (Figure 1). These genes include those of phenylalanine ammonia lyase (Emiliani et al., 2009), which might be involved in defense against microbial pathogens and/or protection against UV, and a transaldolase-like protein (Yang et al., 2014), which also has a specific role in plant defense mechanisms. Additionally, the plant DNA-3-methyladenine glycosylase (*MAG*) gene, which is involved in resistance to a wide variety of DNA damaging agents, contains five introns, and their positions and phases are conserve in many plants including *K. flaccidum* (Fang et al., 2017). However, the *C. braunii MAG* gene has a considerably different gene structure as it contains no introns (Figure 1).

There is no doubt that embryophytes originated from charophytes, however, there is a controversy in some papers on the closest ancestor of embryophytes among taxons of charophytes (Cooper, 2014). Most recent analyses place Coleochaetophyceae or Zygnematophyceae as the closest living cousins of embryophytes. The classes Charophyceae and Klebsormidiophyceae seem to represent earlier evolved taxons, and Chlorokybophyceae and Mesostigmatophyceae are currently considered by the majority of authors as basal phylogenetic groups among charophytes (Delwiche and Cooper, 2015; de Vries et al., 2018; Rensing, 2018). However, it is possible that basal charophytes already populated the land and that important adaptive evolutionary steps in charophytes occurred on land and not in water. It is also important that many extant multicellular charophytes became secondarily aquatic (Harholt et al., 2016). This event in the evolution of charophytes explains findings of genes involved in terrestrial adaptation (stress sensing and stress signal transduction) in their aquatic forms. Moreover, some present-day charophytes among Klebsormidiophyceae and Zygnematophyceae occupy hydro-terrestrial habitats or aero-terrestrial habitats (Karsten et al., 2016; Pierangelini et al., 2017, 2018; Mikhailyuk et al., 2018). These data are in line with the identification of stress genes characteristic for land plants in several classes of algae (Fang et al., 2017). Thus, it can be proposed that 4/1 and some other stress-related genes have appeared in the course of adaptation of Charophyceae ancestor species to land environment and preserved in the present-day genus *Chara* as a remnant genome part.

Evidently, expanding comparative genomics to the most basal charophyte algae, namely classes Chlorokybophyceae and Mesostigmatophyceae, can potentially shed new light on the evolution of 4/1 and other stress-related genes and proteins in water and terrestrial environments. Taken together, our analyses reveal that intronless sequences of charophyte stress-related genes might have emerged by retroduplication in ancestors of Charophyceae and subjected to new intronization steps in early land plants (Figure 1). Nevertheless, this hypothesis does not exclude other evolutionary scenarios that involved no retroduplication events. Assuming potential origin of some plant genes from prokaryotes, repeated exonization-intronization events could be an important evolutionary process in the emergence of plant stress-related genes in Viridiplantae.

## Author Contributions

Both authors have made a substantial, direct, and intellectual contribution to the work, and approved it for publication.

### Conflict of Interest Statement

The authors declare that the research was conducted in the absence of any commercial or financial relationships that could be construed as a potential conflict of interest.
