# Supplementary material for: Emergence of Intronless Evolutionary Forms of Stress Response Genes: Possible Relation to Terrestrial Adaptation of Green Plants
Source: Front Plant Sci. 2019 Feb 7;10:83. doi: 10.3389/fpls.2019.00083 (PMC6374339; doi:10.3389/fpls.2019.00083)
Supplement: Supplementary file 1 [file Image_1.pdf]

Chara braunii (class Charophyceae)

```
MLGEKKVDILEMTSELEREYKQTKAKCRELTAQLAREEVTRKCLEDRCQDLLRTNECLKQVNAECVAMAREQ
--------HHHHHHHHHHHHHHHHHHHHHHHHHHHHHHHHHHHHHHHHHHHHHHHHHHHHHHHHHHHH
------------cccccccCCCCCCCCCCCCCCCCCCCCCCCCCCCCCCCCCCCCCCCCCCC-----------

YDFRKKSKELSIETEAKEDAWRRKERDWEKRVRDLNDELREERRQHKELKDKYLELSSRMEEEMRARRERE
HHHHHHHHHHHHH-HHHHHHHHHHHHHHHHH--HHHHHHHHHHHHHHHHHHHHHHHHHHHHHHHHHHHHHH
------------------------CCCCCCCCCCCCCCCCCCCCCCCCCCCCCCCCCCCCCCCCCC-----

KDWERQLRRLGDDMEDRRRRHEDLVASLEAQVAKTTDEIAVLRQENEAAAKRQQEEQLAADIKIAALLEK
HHHHHHHHHHHHHHHHH---HHHHHHHHHHHHHHHHHHHHHHHHHHHHHHHHHHHHHHHHHHHHHHHHHH
-----------------CCCCCCCCCCCCCCCCCCCCCCCCCCCCCCCCCCCcccccccccccCCCCCCC

LQNADRRGSQLEGDLRKAEHTNTVIRTRYEEQLRNQHPRLIHQVEILKEQVSRLRKERDDLLRDLQF
HHHHHHHHHHHHHHHHHHHHH-HHHHHHHHHHHHHHHHHHHHHHHHHHHHHHHHHHHHHHHHHH---
cccccccccccccc----------------------cccccccCCCCCCCCCCCCCCCCCCCCCC---
```

Spirogyra pratensis (class Zygnematophyceae)

```
MDSLINLLEDIDSIQEDYYGLKNEIHILKGNLAFEENKALAFEKCNSDLILEIDNLKKFHEEELKLINKQYD
-HHHHHHHHHH--HHHHHH----EEEEEE--------HHHHHHHHHHHHHHHHHHHHHHHHHHHHHHHHHHH
---CCCCCCCCCCCCCCCCCCCCCCCCCCCCCCCCCCC----------------------------------

FYAKYKNACETIEKLEDSNKTKLQDEIERRMQAESFSNFEKKQLLEKIGKLEKCVVEGEEKKKSLEFQVAD
HHHHHHHHHHHHHHHH--HHHHHHHHHHHHHHHHHHHHHHHHHHHHHHH---HHHHHHHHHHHHHHHHHHH
-------------------------------------------------------------CCCCCCCCCC

LSSQILELTKKNEEDIMSAEVKFFDELNDVQAKYAEKCEEVNKLEKALTEADKETMASKMKMEEFHRQEG
HHHHHHHHHHHHHHH-------HHHH--HHHHHHHHHHHHHHHHHHH-----HHHHHHHHH-----HHH-
CCCCCCCCCCC---------------cccccccCCCCCCCCCCCCCCCCCCCCC----------------

KNWQMEIFKQKAIQLRKENEELKQKLQLLMPQNASRF
-HHHHHHHHHHHHHHHHH-HHHHHHHHHHH-------
----CCCCCCCCCCCCCCCCCCCCCccccccc-----
```

Anthoceros agrestis (class Anthocerotophyta)

```
MHLTLEGDDRRYATGARYAGHCVAAKCACSASRTRPRFHHAFPYTFHDEVHRNLGCSDLAEDQVHRGTREQV
---EE----------------EEE----------------------HHHHHHH----HHHHHHHHHHHHHHH
-----------------------------------------------------------------------

LSLSACLASEVRRREAAEMRCASLYRDKNRLSTFQKESLLEKAGQVESRAKYYHLVDQVKHLEQHMEERSQ
HHHHHHHHHHHHHHHHHHHHHHHHHHH-HH--HHHHHHHHHHHHHH----HHHHHHHHHHHHHHHHHHHHH
-----------------------------------------------------------------------

VPAVSTTGSQSKHEVQVLELHLRIRGLESELALSQSRAAALVQQVEEFTTSAGSIQCDVQRAKGDLHMKD
HHHHHHHHHHHHHHHHHHHHHHHHHHHHHHHHHHHHHHHHHHHHHHHHHHHHHHHHHHHHHHHHHHHHHH
----------------CCCCCCCCCCCCCCCCCCCCCCCCCCCCccccccc-------------------

KRERDNLARKLETSESANYTLRQELCIAQHQALAATERLEEHLMRQSHSNRHVEAFKRRIMMLRQENEELKRRPQG
HHHHHHHHHHHHHHHHH--HHHHHHHHHHHHHHHHHHHHHHHHHH---HHHHHHHHHHHHHHHHHHHHHHHHH---
----------------------------------------------------CCCCCCCCCCCCCCCCCCCCC---
```

Marchantia polymorpha (class Marchantiophyta)

```
MSPAWDEYFATDPSSPATPFSPNCSASRHSDQVAIRPPQMQNENEILALLGDVDALQKQYDEGLHEIHAVKT
--------------------------------EEE-------HHHHHHHHHHHHHHHHHHHHHHHHHHHHHH
------------------------------------------------------------------------
```

```
LLCVEQKKRKIAETTCIDLEREKQQLLKAQLATYERASEQLDYARKYENVLQLFKGAPENSDRNDKTQDQD
----HHHHHHHHHH-HHHHHHHHHHHHHHHHHHHHHHHHHHHHHHHHHHHHHHHHHHHHH----HHHHHHH
--------ccccccccccccccccccccc------------------------------------------

IDILALDTAKLGFQEHQKTITDLLKRSEAKVGELHKQIMVLREDVTLKECAITALEQQLAQSVTPDTLEM
HHHHHHHHHHHHHHHHHHHHHHHHHHHHHHHHHHHHHHHHHHHHHHHHHHHHHHHHHHHH---HHHHHHH
------------------------------CCCCCCCCCCCCCCCCCCCCCCCCCCCC------------

KLQEAEKRMHDLYREEIEGLRNKLKAEEVNNQKLKSQLEDAHRTLMVSSVNHDDQLRELSANRQADIFKQ
HHHHHHHHHHHHHHHHHHHHHHHHHHHHHHHHHHHHHHHHHHHHHHH---HHHHHHHH--HHHHHHHHHH
---------cccccccCCCCCCCCCCCCCCCCCCCCCCCCCCCC--------------------------

KVLKLRKDNEVLRRQMQQNLK
HHHHHHHHHHHHHHHHH----
-CCCCCCCCCCCCCC------
```

Selaginella moellendorffii (class Isoetopsida)

```
MLPPSEEDMEVLARDLAALEEEFRDAKSEAESLRALLAAETQCRQAAENLCASLEGDKARLMKSQEEFLAQT
-----HHHHHHHHH-HHHHHHHHHHHHHHHHHHHH---HHHHHHHHHHHHHHHHHHHHHHHHHHHHHHHHHH
-cccccccCCCCCCCCCCCCCCCCCCCCCCCCCCCC-------ccccccccccccccccccccc--------

VQQLDYKSKFEVLWQEVNLRSEQDNAMQQEHERAVSTLQEKFQTMEAELNARISSLEKQLRSKESCVIQLE
HHHHHH-HHHHHHHHHHHHHHHHHHH-HHHHHHHHHHHHHHHHHHHHHHHHHHHHHHHHHHHHHHHHHHHH
----------------------------------------------------CCCCCCCCCCCCCCCCCCC

QEIAQLHDSMDILNQNHEERRKRDQTEFQREITALKLELQTAKQANHSLSEQLEKQQQNSLLEKTRLEAQ
HHHHHHHHHHHHHHHHHHHHHHHHHHHHH-HHHHHHHHHHHHHHHHHHHHHHHHHHHHHHHH-HHHHHHH
CCCCCCCCCCCCCCCC---------------CCCCCCCCCCCCCCCCCCCCCCCCCCCC-----------

IQDLSSNWQVGALKQKLMKLRKENEDLKRRRF
HH---HHHHHHHHHHHHHHHHH--HHHHH---
---------CCCCCCCCCCCCCCCCCCCCC--
```

Ophioglossum vulgatum (class Polypodiopsida)

```
MAPSDEELSSLLRDFEALYQEYKDCASEGAVMKSKLNIEVQKREASDHHCAELEKENQRLLKIQGEILSLMS
----HHHHHHHHH-HHHHHHHHHHHHHHHHHHHH---HHHHHHHHHHH-------HHHHHHHH--HHHHHHH
----------------------------------------------------CCCCCCCCCCCCCCCCCCCC
NQIEYRSKYEVLSNQFKLVEEQLKENDKEHKIKLDIIAEENIKKVKQLDNEIRQAQEQMALKDVAMAQLEE
-----------HHHH-----HHHHHHHHHHHHHHH----HHHHHHHHHHHHHHHHHHHHH---HHHHH-HH
C------------------------------------CCCCCCCCCCCCCCCCCCCCCCCCCCCCCCCCCC
EITHFRSMIDTLNRSLQQTHIDAEKKYWHDIDELQKQLHMEMQEKCKLQERIQQLENSSTVARMKYEEQL
HHHHH--HHHHHHHHHHHHHHHHHHHHHH--H-HHHHHHHHHHHHHHHHHHHHHHH---------HHHHH
CCCCCCCCCCCCCCCCCCCCCC--------CCCCCCCCCCCCCCCCCCCCCCCCCCCC------------
RECSSNRHAEAFKQKIMRLRKENEELKRRLLCSV
HH---HHHHHHHHHHHHHHHHHHHHHHHHH----
--------CCCCCCCCCCCCCCCCCCCCC-----
```

Cephalotaxus_harringtonia (subclass Pinidae)

```
MDSLLDDFDHLHKEYNDGILEIQSLKEKYAIEVKRCEALKVTCIGLKKDKERLMKSHTETLNKLTEQLDYHT
--HHHHHHHHHHHHHHHHHHHHHHHH----HHHHHHHHHHHH-------HHHHHHHHHHHHHHHHHHHH-HH
----------------------------------------------ccccccccccccccccccccc-----
```

```
KYQSALDQVDTMKEEILIKEKKHETTLESLETKHQKKILELQKLIRTTSECLQEKHQEEIGKLQMQIRYNQ
HHHHHHHHHHHHHHHHHH-HHHHHHHHHHHHHHHHHHHHHHHHHHHHHHHHH-HHHHHHHHHHHHHHHHHH
--------------------------ccccccccccccccccccccc------------------------

TQLSVKEEIIAQLQIELADCEAMTDALKDRLDIIEHNAKLKYEHDMQELRDKLQLELEAKNKLHEELKNV
HHHHHHHHHHHHHHHHHHHHHHHHHHHHHHHHHHHHHHHHHHHHHHHHHHHHHHHHHHHHHHHHHHHHHH
---------ccccccccccccccccccccc--------cccccccCCCCCCCCCCCCCCCCCCCCCcccc

KHTLLINKMKYEEQLRDSSSNRQIDMFKQKIMKHRKENEDLRRQLLSTRQ
HHHHHHH--HHHHHHHHHHHHHHHHHHHHHHHHHHHHHHHHHHHH-----
-----------------------CCCCCCCCCCCCCCCCCCCCC------
```

Nt-4/1 (subclass Asterids)

```
MAATTDEAMESLLSSFDQIYDEFKNGAVEIQTLQADYITGSTKREALEFTVQRLQSENDRLRKLYAESLNTL
-----HHHHHHHHH-HHHHHHHHHHHHHHHHHHH-----HHHHHHHHHHHHHHHHHHHHHHHHHHHHHHHHH
-------------------------------------------CCCCCCCCCCCCCCCCCCCCCccccccc-

AYKIESHSSCQSLKEDLKGVHHEFLQKENEYIRTIESLKGDHAERIQELESQIRRYQTEDAVNKATINQLR
HHHHHHHHHHH-HHHHHHHHHHHHH--HHHHHHHHHHHHHHHHHHHHHHHHHHHHHHHHHHHHHHHHHHHHH
--------------------------------------CCCCCCCCCCCCCCCCCCCCC-------CCCCC

HDLVVHRSQMEALKRNLDQVSAEVDSRYHYEIQGLKDSLLVEQEEKNELNKRLRDMEKELFISRTKLVEY
HHHHHHHHHHHHHHHHHHHHHHHHHHHH--HHHHHHHHHHHHHHHHHHHHHHHHHHHHHHHHHHHHHHHH
CCCCCCCCCCCCCCCCCCCCCCC--------CCCCCCCCCCCCCCCCCCCCCCCCCCCCccccccc----

QQDSTSSQHMHTLKQKIMKLRKENEVLKRQIHERKEF
H----HHHHHHHHHHHHH--HHHHHHHHHHHHH----
---------CCCCCCCCCCCCCCCCCCCCC-------
```

Supplementary Figure 1. Secondary structures of some selected 4/1-like proteins according to www.compbio.dundee.ac.uk/jpred/. The α-helical (H) and coiled-coil (C) protein regions are shown. *Chara braunii* gene CBR_g25877 has 30% identity (e-value=1e−04) to the partial 4/1-like protein of *S. pratensis*, 28% identity (e-value=3e−04) to 4/1 protein of *M.polymorpha*, 29% (e-value=3e−04) to 4/1 protein of *S. moellendorffii*, 27% (e-value=2e−06) to 4/1 of *C. harringtonia*, 25% (e-value=1e−07) to 4/1 of *O. vulgatum* and 27% (e-value=0.005) to Nt-4/1 of *Nicotiana tabacum*.
